# Reassessment of the prognostic value of the International Prognostic Index and the revised International Prognostic Index in patients with diffuse large B-cell lymphoma: A multicentre study

**DOI:** 10.3892/etm.2012.607

**Published:** 2012-06-12

**Authors:** HONG-HUI HUANG, FEI XIAO, FANG-YUAN CHEN, TING WANG, JUN-MIN LI, JIAN-MIN WANG, JUN-NING CAO, CHUN WANG, SHAN-HUA ZOU

**Affiliations:** 1 Department of Hematology, Renji Hospital;; 2 Department of Hematology, Ruijin Hospital, Shanghai Jiaotong University School of Medicine;; 3 Department of Hematology, Changhai Hospital, Second Military Medical University;; 4 Department of Medical Oncology, Fudan University Shanghai Cancer Center;; 5 Department of Hematology, Shanghai First People’s Hospital affiliated to Shanghai Jiaotong University;; 6 Department of Hematology, Zhongshan Hospital, Fudan University, Shanghai, P.R. China

**Keywords:** International Prognostic Index, diffuse large B-cell lymphoma, rituximab

## Abstract

The International Prognostic Index (IPI) is a widely accepted model that is used to predict the prognosis of patients with diffuse large B-cell lymphoma (DLBCL) who are treated using chemotherapy. However, the prognostic value of the IPI has been a focal point of debate in the immunochemotherapy era. The aim of this study was to reassess the value of the IPI and revised IPI (R-IPI) in a Chinese population. A multicentre retrospective analysis of DLBCL patients who were treated with cyclophosphamide, doxorubicin, vincristine and prednisone (CHOP)-like chemotherapy alone or chemotherapy plus rituximab (R-CHOP-like) was performed. The prognostic values of IPI and R-IPI at the time of diagnosis with respect to overall survival (OS) and progression-free survival (PFS) were evaluated. Among the 438 patients in the study, 241 received a CHOP-like regimen and 197 patients received an R-CHOP-like regimen. Although the IPI remained predictive for the CHOP-like group, it failed to distinguish between the various prognostic categories in the R-CHOP-like group. Notably, redistribution of the IPI factors into R-IPI factors identified three discrete prognostic groups with significantly different outcomes in both the CHOP-like and R-CHOP-like groups. In the R-CHOP-like group, these three risk groups, very good, good and poor, had distinctly different 3-year PFS rates of 96, 84.3 and 67.5% (P=0.001), and 3-year OS rates of 96, 87.6 and 71.1% (P=0.003), respectively. Our study demonstrates the power of the R-IPI as a simplified and more clinically relevant predictor of disease outcome than the standard IPI in DLBCL populations in the rituximab era. Therefore, the R-IPI merits further study in a larger population-based prospective study.

## Introduction

Diffuse large B-cell lymphoma (DLBCL) is the most common type of non-Hodgkin’s lymphoma (NHL). Recently, a large epidemiological study was performed in the Chinese population. Although the official data have yet to be published, the preliminary results and other clinicopathological studies have shown that DLBCL accounts for approximately 30–40% of new NHL cases in China (1,2). Current insight into the pathogenesis of DLBCL and its highly variable outcome suggest that DLBCL is a heterogeneous group of lymphomas with various genetic abnormalities, clinical characteristics and prognosis, as opposed to a single clinicopathological entity (3).

During the past several decades, the cyclophosphamide, doxorubicin, vincristine and prednisone (CHOP) chemotherapy regimen has been the gold standard of DLBCL therapy. However, the cure rate that is achieved using standard chemotherapy alone is only 30–40% (4,5). Recently, the addition of rituximab to the conventional CHOP regimen (R-CHOP) has led to a marked improvement in survival in previously untreated elderly (older than 60 years of age) and young (60 or younger) DLBCL patients. These results have been confirmed by the Groupe d’Etude des Lymphomes de l’Adulte study (GELA/LNH-98-5) (6) and the MabThera International Trial (MInT) (7).

Although considerable progress in the treatment of DLBCL has been made in the era of rituximab, a significantly variable outcome remains for a substantial minority of patients who are not cured. Therefore, a well-validated and accurate prognostic system is required to serve as the basis for risk-adjusted therapy for DLBCL. Such a system would allow clinicians to rapidly and easily stratify patients with various prognoses and provide a more individualised therapy.

The International Prognostic Index (IPI) was introduced by Shipp and the International Non-Hodgkin’s Lymphoma Prognostic Factors Project in the 1990s (8,9) and has become a widely accepted prognostic index for patients with aggressive lymphomas. In this system, the patient age, tumour stage, serum lactate dehydrogenase concentration, performance status and number of extranodal disease sites all serve to identify four discrete risk groups with varying 5-year overall survival rates that range from 26 to 73%.

However, the IPI system is an individual case-based prognostic factor for the analysis of CHOP-like regimens. In recent studies, the question of whether (and to what extent) the prognostic index should be changed in the context of novel immunochemotherapy strategies has been intensely debated. More recently, Sehn *et al* investigated this issue by analysing the role of the IPI in a register-based retrospective study of 365 patients who were treated with R-CHOP (10). Their results suggested that the revised International Prognostic Index (R-IPI) predicts patient outcome more reliably than the standard IPI for DLBCL patients who are treated with R-CHOP. The R-IPI is a redistribution of the original IPI factors rather than an identification of new prognostic markers. Using a scoring system with the same adverse parameters, three discrete prognostic groups (0=very good; 1 or 2=good; and 3, 4, or 5=poor) can be identified as having significantly different outcomes.

To date, most of the reports regarding the IPI or R-IPI have been limited to Western countries. Therefore, we conducted a multicentre retrospective analysis of Chinese DLBCL patients who were treated with CHOP-like chemotherapy alone or R-CHOP-like chemotherapy to reassess whether the IPI system, which is a well-known paradigm for risk assessment, is applicable to Chinese DLBCL populations and therefore still has value in the immunochemotherapy era. We also assessed whether the R-IPI system discriminates more between patients with various prognoses. To the best of our knowledge, this is the first report of a multicentre study in an unselected Chinese population of DLBCL patients.

## Materials and methods

### Patient characteristics

This study was a retrospective analysis of newly diagnosed DLBCL patients who were treated at six participating hospitals between 1997 and 2008, and were evaluated by the Shanghai Lymphoma Research Group (SLRG). The histological diagnoses were reviewed and classified according to the WHO classification system. The clinical and demographic data were also reviewed. Patients were selected for inclusion in this study based on the following criteria; a histologically confirmed diagnosis of CD20-positive DLBCL, previously untreated, aged 18 or older, no primary or secondary central nervous system involvement, no cardiac contraindication to anthracycline treatment, no human immunodeficiency virus infection and no severe co-morbid illness that precluded the treatment of lymphoma. Informed consent was obtained from every patient. The study was approved by the ethics-review committee of every participating centre.

### Treatment and efficacy evaluation

All patients were treated with a CHOP-like regimen ± rituximab as a first-line therapy. In the R-CHOP-like group, the dosage and schedule of rituximab was 375 mg/m^2^ on day 0 of each cycle for a minimum of four cycles. The treatment cycle was 21 days in length. The patients who experienced neutropenia received granulocyte colony-stimulating factor (G-CSF); if the neutropenia persisted until the next cycle, the following cycle was postponed by up to two weeks. The efficacy evaluation was performed upon the completion of the treatment. The response of the lymphoma was classified as a complete response (CR), a partial response (PR), a stable disease (SD), a progressive disease (PD), or a relapse (R) in accordance with the International Workshop Criteria (11).

### Statistical methods

The analysis was based on the follow-up information that was available by May 1 2010. Progression-free survival (PFS) was calculated from the date of the diagnosis until either documented disease progression or the date that the patient was last known to be alive for patients who succumbed to causes that were unrelated to the lymphoma or its treatment. Overall survival (OS) was calculated from the date of the diagnosis until mortality, regardless of the cause. PFS and OS were assessed using the Kaplan-Meier method and were compared between the groups using the log-rank test. The clinical characteristics and efficacy evaluations were compared between the subgroups using the χ^2^ test. All P-values were calculated as two-sided, and P<0.05 was considered to indicate a statistically significant result. The data were analysed using the Statistical Software Package for the Social Sciences (SPSS version 13.0 for Windows; SPSS Inc., Chicago, IL, USA).

## Results

### Patient characteristics

A total of 438 patients who met the inclusion criteria were identified, among which 241 were treated with a CHOP-like regimen alone and 197 patients were treated with an R-CHOP-like regimen. The majority of the patients (96.6%) received an anthracycline-based regimen, and the remaining patients (3.4%) were treated with mitoxantrone-COP. The median age at diagnosis was 50 years (range, 18–83 years) and the male:female ratio was 1.06:1. Overall, 75.6% of the patients completed 6–8 cycles of chemotherapy. The median follow-up was at 34 months (range, 3–145 months), and 106 mortalities were reported by the time of the last follow-up; among these mortalities, 73 and 33 occurred in the CHOP-like and R-CHOP-like groups, respectively. The clinical characteristics and distributions of the individual IPI factors at the time of diagnosis are listed in Table I. The baseline patient characteristics were matched for age, gender, number of treatment cycles and prognostic factors of the IPI between the two groups.

### Patient outcome according to the standard IPI strata

**Figure 1:**
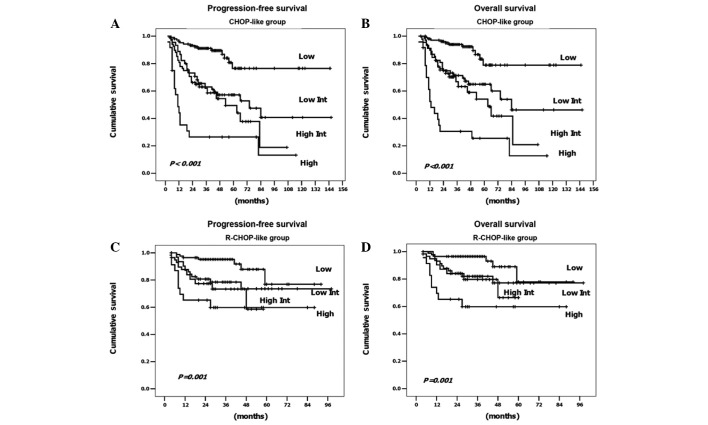
Outcome according to the standard International Prognostic Index (IPI) for (A) progression-free survival and (B) overall survival in the 241 patients treated with chemotherapy alone and for (C) progression-free survival and (D) overall survival in the 197 patients who were treated with chemotherapy plus rituximab. CHOP, cyclophosphamide, doxorubicin, vincristine and prednisone; R-CHOP, rituximab plus the conventional CHOP regimen.

The treatment outcomes based on the standard IPI are summarised in Table II. Within the group of 241 patients who were treated with CHOP-like alone, the standard IPI score was highly predictive of outcome and discriminated well between the four groups. The 3-year PFS rates ranged from 26.5 to 91.1% (P<0.001) and the 3-year OS rates ranged from 30.6 to 93.9% (P<0.001). Within the group of 197 patients who were treated with R-CHOP-like, the IPI score remained predictive. The 3-year PFS rates ranged from 59.8 to 95.2% (P=0.001), and the 3-year OS rates ranged from 59.8 to 96.4% (P=0.001). However, the IPI score no longer distinguished between the four risk groups; instead, the low/low-intermediate risk group and the high-intermediate/high risk group showed clearly overlapping curves (Fig. 1).

### Patient outcome according to the R-IPI strata

**Figure 2:**
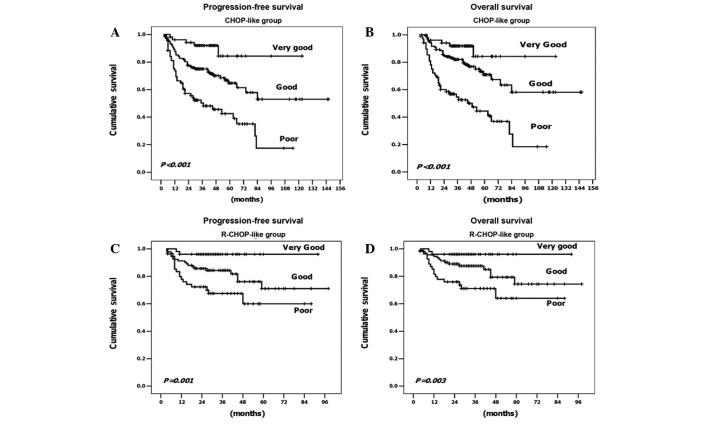
Outcome according to the revised International Prognostic Index (R-IPI) for (A) progression-free survival and (B) overall survival in the 241 patients treated with chemotherapy alone and for (C) progression-free survival and (D) overall survival in the 197 patients who were treated with chemotherapy plus rituximab. CHOP, cyclophosphamide, doxorubicin, vincristine and prednisone; R-CHOP, rituximab plus the conventional CHOP regimen.

To determine the efficacy of the R-IPI, the patients were redistributed into three groups containing 0, 1–2 and 3–5 risk factors, thereby generating the following three new prognostic groups; very good, good and poor, respectively. The PFS and OS of the CHOP-like and R-CHOP-like patients based on their R-IPI score are displayed in Table II and Fig. 2. We observed three discrete curves that represent the three prognostic groups. In the group of patients who were treated with an R-CHOP-like regimen, the three risk groups had distinctly different 3-year PFS rates of 96, 84.3 and 67.5% (P=0.001) and 3-year OS rates of 96, 87.6 and 71.1% (P=0.003), respectively. In the group of patients who did not receive rituximab, similar results were observed, except for reduced PFS and OS in the CHOP-like group compared with the R-CHOP-like group (this was particularly true for the patients in the poor prognostic group). Thus, the R-IPI was prognostically relevant for both PFS and OS in the DLBCL patients who were treated with either an R-CHOP-like or CHOP-like regimen.

## Discussion

Over the past decade, considerable effort has been expended in the search for predictive and prognostic information to use for risk stratification in clinical settings. Since its introduction in 1993, the IPI system has become a well-known, convenient and useful prognostic index for DLBCL patients. Among the various novel prognostic models that contain numerous promising biomarkers, including those of cellular origin (such as germinal centre B cell and activated B cell types) and immunoexpression patterns (including p53, Ki67, Bcl-2, Bcl-6, CD10 and CD5), none has achieved the required level of acceptance to be routinely used for risk stratification of DLBCL patients (12–16).

Since a number of studies have demonstrated that the introduction of new therapies is able to change the predictive value of established prognostic systems, the relevance of the prognostic markers varies depending on the geographic region and patient ethnicity, due to the various baseline clinical characteristics of these patients. Thus, revalidation of these systems is essential. The goal of the present study was to reassess the current perceived value of the standard IPI system and to determine whether the R-IPI system reliably discriminates between patients with different survival outcomes in the Chinese population.

Our data demonstrate that among patients who were treated with a CHOP-like regimen alone, the standard IPI score was able to predict the outcome and discriminate clearly among the four groups. However, in the patients who were treated with an R-CHOP-like regimen, although the score remained predictive, the standard IPI could not distinguish between the patients who were at low/low-intermediate risk and high-intermediate/high risk in the Kaplan-Meier survival curve. The groups produced closely overlapping curves according to the standard IPI strata and this is consistent with the results of previous clinical studies by Sehn *et al* (10) and Bari *et al* (17).

In addition, in the present study, we attempted to apply the R-IPI to the patients who were treated with a CHOP-like or R-CHOP-like regimen. Upon redistribution of the IPI factors into an R-IPI, a more clinically useful prediction of outcome was observed in both groups of patients treated with or without rituximab. In the CHOP-like group, three risk groups were identified based on the R-IPI strata; very good, good and poor; with 3-year PFS rates of 91.8, 75.6 and 50.2%, respectively, and 3-year OS rates of 91.8, 82.7 and 54.7%, respectively. Notably, the results were particularly significant from the patients who received the R-CHOP-like regimen. Three clear curves representing prognostic groups were observed. Of these patients, 25% were classified into the very good prognostic group, with a greater than 96% chance of 3-year PFS and OS. Therefore, according to a series of randomised studies, R-CHOP is now preferable to CHOP as the gold standard of therapy for this favourable prognostic group of patients with DLBCL. However, despite the extremely favourable outcome, care must be taken to avoid excessive toxicity that can result from unnecessary treatments. Almost 50% of the patients were classified in the good prognostic group and had 3-year PFS and OS rates of 84.3 and 87.6%, respectively. To improve the outcome for this less-favourable subgroup of patients, randomised clinical trials may be required. Among the poor risk group, which comprised 28% of the cases, the 3-year PFS and OS rates were only 67.5 and 71.1%, respectively. To improve the prognosis for these patients, early intervention with innovative approaches or high-dose chemotherapy followed by autologous stem cell rescue should be considered. In a group of 94 DLBCL patients, Vitolo *et al* (on behalf of the Italy GIMURELL group) reported the following impressive results for dose-dense and high-dose chemotherapy plus rituximab together with autologous stem cell transplantation; in good-risk patients, according to the R-IPI, the 4-year OS rate was estimated at 87% and the most significant finding was that these positive results persisted for poor-risk patients with a 4-year OS rate that was estimated at 73% (18). These encouraging data indicate that patients with a poor prognosis should benefit from this treatment strategy.

We noted that the addition of the R-IPI to the prognosis of DLBCL patients in the rituximab era has remained controversial, as demonstrated by certain recent reports. An analysis performed by Ziepert *et al* (19), which involved 1,062 patients with DLBCL who were accrued from three prospective trials [the MinT, RICOVER-60 (20) and MegaCHOEP (21) trials], confirmed the prognostic relevance of the standard IPI score for PFS, event-free survival and OS endpoints. Thus, they concluded that the standard IPI is still valid for patients with DLCBL in the R-CHOP era. However, Tay *et al* (22) recently noted certain weaknesses in the study. First, different chemotherapy regimens were used in the three trials from which the data were collected. Second, their data set was an under-representation of young high-risk patients. Only 55 (5.2%) of the young patients with two or more risk factors were included in the analysis. In another study, Advani *et al* (23) evaluated the performance of the standard IPI and the following modifications; age adjusted (AA)-IPI, R-IPI and an elderly IPI with an age cut-off of 70 years (E-IPI) in patients over 60 years of age who were treated with R-CHOP. They found that the R-IPI did not identify a highly favourable risk group, thus minimising its usefulness in this population, whereas the IPI and AA-IPI were considered to be useful tools for outcome prediction in patients over the age of 60 who were treated with R-CHOP. However, this conclusion applies only to elderly patients and is less appropriate for other DLBCL populations. Notably, in our study, our data support the conclusion that the R-IPI has a strong prognostic relevance for PFS and OS among patients who are treated with or without rituximab. To the best of our knowledge, this is the first multicentre study analysing the impact of the R-IPI in an unselected Chinese population of DLBCL patients.

In conclusion, this multicentre study provides insights for analysing prognostic factors in DLBCL patients. Our data indicate that the standard IPI is applicable to Chinese DLBCL populations and remains predictive for the chemotherapy group. However, adjustments are required for a more precise prognostic prediction in the immunochemotherapy era. The R-IPI, which is a redistribution of the original IPI factors, may serve as a simplified and more clinically relevant predictor of outcome than the standard IPI, and the use of the R-IPI may aid the identification of poor prognostic subgroups of newly diagnosed DLBCL patients. Since the current study was not a randomised clinical trial, a larger population-based prospective study and meta-analysis are highly recommended for further validation of our conclusions. New therapeutic strategies and early interventions are required to improve the outcome of DLCBL in the R-IPI poor prognostic group.

## Figures and Tables

**Table I:** Patients and disease characteristics.

Characteristic	R-CHOP-like (n=197)	CHOP-like (n=241)
Age, years, median (range)	55 (18–80)	54 (18–83)
Male	98 (50%)	136 (56%)
Median observation time, months (range)	31 (4–97)	39 (3–145)
IPI factors		
Age, years		
>60	59 (30%)	83 (34%)
≤60	138 (70%)	158 (66%)
Performance status		
>1	42 (21%)	53 (22%)
≤1	155 (79%)	188 (78%)
Lactate dehydrogenase		
>normal	77 (39%)	81 (33%)
≤normal	120 (61%)	160 (67%)
Stage		
III–IV	107 (54%)	134 (56%)
I–II	90 (46%)	107 (44%)
Extranodal sites		
>1	48 (24%)	74 (30%)
≤1	149 (76%)	167 (70%)

IPI, International Prognostic Index; CHOP, cyclophosphamide, doxorubicin, vincristine and prednisone; R-CHOP, rituximab plus the conventional CHOP regimen.

**Table II:** Outcome according to the standard IPI and the revised-IPI.

		R-CHOP-like			CHOP-like		
Risk group	No. of IPI factors	No. (%)	3-year PFS	3-year OS	No. (%)	3-year PFS	3-year OS
Standard IPI							
Low	0, 1	84 (43)	95.2%	96.4%	105 (44)	91.1%	93.9%
Low-intermediate	2	58 (29)	78.5%	81.9%	67 (28)	63.9%	72.4%
High-intermediate	3	32 (16)	73.0%	79.7%	45 (19)	62.0%	66.8%
High	4, 5	23 (12)	59.8%	59.8%	24 (10)	26.5%	30.6%
P-value			0.001	0.001		<0.001	<0.001
Revised IPI							
Very good	0	50 (25)	96.0%	96.0%	51 (21)	91.8%	91.8%
Good	1, 2	92 (47)	84.3%	87.6%	121 (50)	75.6%	82.7%
Poor	3, 4, 5	55 (28)	67.5%	71.1%	69 (29)	50.2%	54.7%
P-value			0.001	0.003		<0.001	<0.001

PFS, progression-free survival; OS, overall survival; IPI, International Prognostic Index; CHOP, cyclophosphamide, doxorubicin, vincristine and prednisone; R-CHOP, rituximab plus the conventional CHOP regimen.
